# Reprogramming Metabolic Flux in *Escherichia Coli* to Enhance Chondroitin Production

**DOI:** 10.1002/advs.202307351

**Published:** 2023-12-25

**Authors:** Chunlei Zhao, Xiaomin Li, Liang Guo, Cong Gao, Wei Song, Wanqing Wei, Jing Wu, Liming Liu, Xiulai Chen

**Affiliations:** ^1^ State Key Laboratory of Food Science and Resources Jiangnan University Wuxi 214122 China; ^2^ International Joint Laboratory on Food Safety Jiangnan University Wuxi 214122 China; ^3^ School of Life Sciences and Health Engineering Jiangnan University Wuxi 214122 China

**Keywords:** chondroitin, metabolic engineering, metabolic flux, molecular valve

## Abstract

Reprogramming metabolic flux is a promising approach for constructing efficient microbial cell factories (MCFs) to produce chemicals. However, how to boost the transmission efficiency of metabolic flux is still challenging in complex metabolic pathways. In this study, metabolic flux is systematically reprogrammed by regulating flux size, flux direction, and flux rate to build an efficient MCF for chondroitin production. The ammoniation pool for UDP‐GalNAc synthesis and the carbonization pool for UDP‐GlcA synthesis are first enlarged to increase flux size for providing enough precursors for chondroitin biosynthesis. Then, the ammoniation pool and the carbonization pool are rematched using molecular valves to shift flux direction from cell growth to chondroitin biosynthesis. Next, the adaptability of polymerization pool with the ammoniation and carbonization pools is fine‐tuned by dynamic and static valve‐based adapters to accelerate flux rate for polymerizing UDP‐GalNAc and UDP‐GlcA to produce chondroitin. Finally, the engineered strain *E. coli* F51 is able to produce 9.2 g L^−1^ chondroitin in a 5‐L bioreactor. This strategy shown here provides a systematical approach for regulating metabolic flux in complex metabolic pathways for efficient biosynthesis of chemicals.

## Graphical abstract

In this study, metabolic flux is systematically reprogrammed to build an efficient microbial cell factory for chondroitin production by regulating flux size, flux direction, and flux rate using static valve, dynamic positive valve, and dynamic negative valve. It offers a new insight for synthesizing other valuable chemicals.

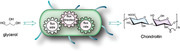

## 1 Introduction

Microbial cell factories (MCFs) are widely used as a sustainable tool for the production of pharmaceutical, biofuel, and food, which provides a good alternative to realize fossil energy substitution and eliminate the dependence on animal and plant raw materials.^[^ ^1^ ^]^ To improve the efficiency of MCFs, many metabolic engineering strategies have been developed, such as static regulation methods and dynamic control tools, to regulate metabolic flux for the biosynthesis of chemicals.^[^ ^2^ ^]^ Although these strategies have been successfully utilized to engineer metabolic pathways for biosynthesizing short‐chain molecules such as *N*‐acetylglucosamine,^[^ ^3^ ^]^ bioethanol,^[^ ^4^ ^]^ and shikimate,^[^ ^5^ ^]^ there are still challenges in remaking complex metabolic pathways for the biosynthesis of long‐chain molecules such as colanic acid, bioengineered heparin, and chondroitin sulfate.^[^ ^6^ ^]^

To engineer complex metabolic pathways in MCFs, four types of strategies have been put forward as a way for fine‐grained regulation of metabolic flux. i) Pushing strategies are used to push substrates utilization or precursors accumulation. In the process of hyaluronic acid biosynthesis, the biosynthetic pathways for accumulating its precursors UDP‐GlcA and UDP‐GlcNAc were screened by introducing multiple pathway enzymes from different microorganisms and further optimized by combinatorially expressing *cg*UgdA2 (UDP‐glucose‐6‐dehydrogenase) from *Corynebacterium glutamicum* and *pt*GlmS (L‐glutamine‐D‐fructose‐6‐phosphate aminotransferase) from *Pichia pastoris*.^[^ ^7^ ^]^ The resulting strain *C. glutamicum* CgspH‐2 realized a significant increase in hyaluronic acid production up to 5.4 g L^−1^, which was 2.6‐fold higher than that of control strain *C. glutamicum* spHasA. To enhance the production of chondroitin, the accumulation of precursors UDP‐GalNAc and UDP‐GlcA was enhanced by co‐expressing GlmS and Ugd. The chondroitin production was up to 7.4 g L^−1^ in *C. glutamicum* CGCH07, which was 9.6‐fold higher than that of *C. glutamicum* CGCH03.^[^ ^8^ ^]^ ii) Driving strategies are applied to drive intermediate metabolites transmission from substrates or precursors to final products. In the process of fructosylated chondroitin biosynthesis, substrate channeling was constructed to improve the transmission efficiency from glycerol to UDP‐GalNAc and UDP‐GlcA by constructing fusion proteins GlmM‐GlmS (phosphoglucosamine mutase and L‐glutamine‐D‐fructose‐6‐phosphate aminotransferase) and GalU‐pgm (glucose‐1‐phosphate uridylyltransferase and phosphoglucomutase).^[^ ^9^ ^]^ The resulting strain *E. coli* ZQ25 realized a significant increase in fructosylated chondroitin production up to 8.43 g L^−1^. iii) Blocking strategies are adopted to reduce the loss of metabolic flux originated from byproducts formation or cell growth. In the process of *N*‐acetylneuraminic acid (NeuAc) biosynthesis, NeuAc synthesis and cell growth in *B. subtilis* was decoupled by a non‐canonical amino acids (ncAA)‐dependent translation system to control transcriptional regulatory of cell wall metabolism (*walR*).^[^ ^10^ ^]^ The resulting strain *B. subtilis* NABd8 achieved a significant increase in NeuAc production up to 4.72 g L^−1^, which was 2.2‐fold higher than that of control strain *B. subtilis* NAB. iv) Pulling strategies are utilized to transport metabolites into organelles or bioproducts out of cells. During the production of fructosylated chondroitin, transporters *kpsE* and *kpsM* were up‐regulated to promote its transport from inside to outside of the cell by enhancing the expression of *slyA*. The resulting strain *E. coli* ThslyA reached 1.0 g L^−1^ fructosylated chondroitin, which was 85% higher than that of wild‐type *E. coli*.^[^ ^11^ ^]^ Based on these “Push‐Drive‐Block‐Pull” strategies, complex metabolic pathways have been engineered and optimized for the production of long‐chain molecules, but its production efficiency is still limited, possibly due to the fact that these strategies mainly focus on regulating metabolic flux, but ignoring the transmission characteristics of metabolic flux. Thus, the reciprocal effect of “Push‐Drive‐Block‐Pull” strategies on metabolic flux restricts the efficiency of MCFs.

Chondroitin is the characteristic long‐chain molecule, glycosaminoglycan, which is widely used in cancer treatment, biomaterials, and antiviral therapy.^[^ ^12^ ^]^ In this study, to systematically enhance the efficiency of MCFs, we classified metabolic flux to flux size, flux direction and flux rate according to its transmission characteristics. Based on this, we designed a systematic method for reprogramming metabolic flux in complex metabolic pathways through regulating flux size, flux direction, and flux rate. Flux size was enlarged to supply enough precursors UDP‐GlcA and UDP‐GalNAc for chondroitin production. Then, flux direction was shifted from cell growth to chondroitin biosynthesis using molecular valves. Next, flux rate was accelerated for polymerizing UDP‐GalNAc and UDP‐GlcA to produce chondroitin by dynamic and static valve‐based adapters. Finally, the engineered strain *E. coli* F51 produced 9.2 g L^−1^ chondroitin in a 5‐L bioreactor.

## 2 Results

### 2.1 Constructing a Microbial Reactor for Chondroitin Synthesis

**Figure 1:**
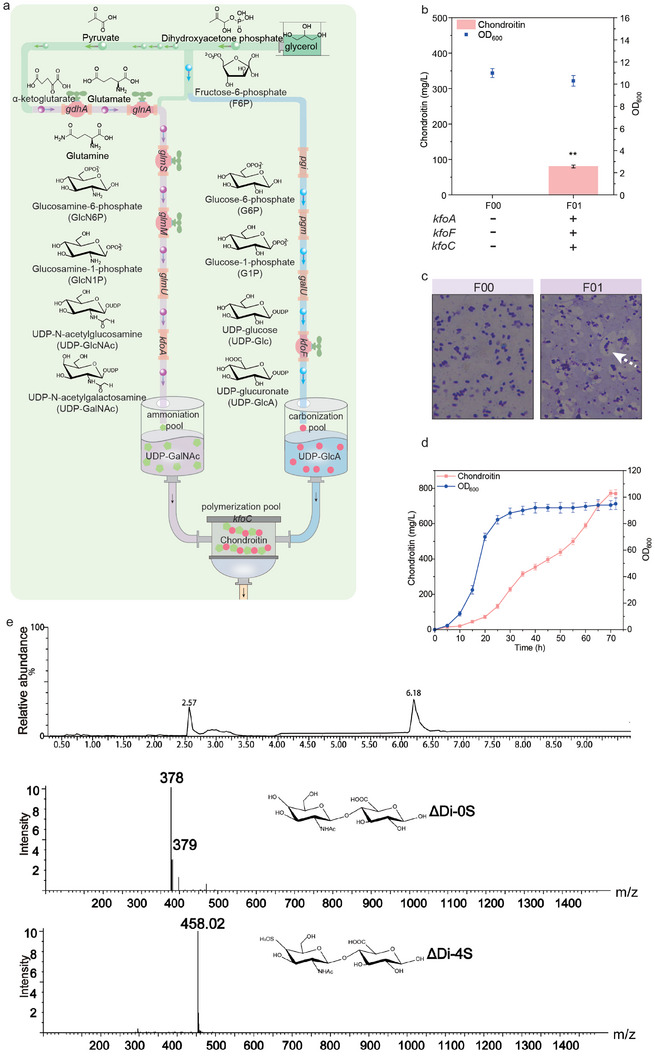
Constructing a microbial reactor for chondroitin synthesis. a) The biosynthetic pathway for chondroitin in *E. coli*. *pgi*, glucose‐6‐phosphate isomerase; *pgm*, phosphoglucomutase; *galU*, glucose‐1‐phosphate uridylyltransferase; *kfoF*, UDP‐glucose dehydrogenase; *gdhA*, glutamate dehydrogenase; *glnA*, glutamine synthetase; *glmS*, L‐glutamine‐D‐fructose‐6‐phosphate aminotransferase; *glmM*, phosphoglucosamine mutase; *glmU*, UDP‐*N*‐acetylglucosamine pyrophosphorylase/Glucosamine‐1‐phosphate *N*‐acetyltransferase; *kfoA*, UDP‐*N*‐acetylglucosamine 4‐epimerase; *kfoC*, chondroitin polymerase. b) Chondroitin production in strain *E. coli* F01. c) Determining the biosynthesis of chondroitin with the crystal violet‐copper sulfate staining method with an optical microscope at ×100 magnification. d) Producing chondroitin by strain *E. coli* F01 in 5‐L fermentor. e) Mass spectrometry to identify chondroitin and its derivative chondroitin sulfate A. ΔDi‐0S, chondroitin disaccharide; ΔDi‐4S, CSA disaccharide. The data were presented as mean values ± SD from three independent biological replicates (n = 3). Statistical significance was evaluated using a two‐tailed Student's t‐test, and significance levels were denoted as ^*^ *p* <0.05, ^**^ *p* <0.01, ^***^ *p* <0.001.

*Escherichia coli* BL21 STAR (DE3) could not service as a microbial reactor for chondroitin biosynthesis, due to lack of three pathway enzymes in the biosynthesis pathway of chondroitin, that is, chondroitin UDP‐glucose‐4‐epimerase (KfoA), UDP‐glucose dehydrogenase (KfoF), and chondroitin polymerase (KfoC) (Figure S1, Supporting Information).^[^ ^13^ ^]^ Based on this pathway, *E. coli* reactor was divided into three modules in vivo: i) the ammoniation pool for UDP‐GalNAc synthesis by introducing the heterologous *kfoA* gene, ii) the carbonization pool for UDP‐GlcA synthesis by expressing the heterologous *kfoF* gene, and iii) the polymerization pool for polymerizing UDP‐GalNAc and UDP‐GlcA to chondroitin by transplanting the heterologous *kfoC* gene (**Figure** **1**a).

To construct *E. coli* reactor to produce chondroitin, we introduced *kfoA*, *kfoF*, and *kfoC* from *E. coli* K4 into *E. coli* BL21 STAR (DE3). The resulting strain *E. coli* F01 produced 80.1 mg L^−1^ chondroitin (Figure 1b), which was confirmed by mass spectrometry (Figure 1e) and staining (Figure 1c). Further, the purified chondroitin was sulfated by 4‐O sulfotransferase (C4ST), and we found that chondroitin sulfate A (CS‐A) was formed (Figure 1e; Figures S2,S3, Supporting Information).^[^ ^14^ ^]^ These results indicated that the de novo biosynthesis pathway of chondroitin was constructed in *E. coli* successfully, suggesting that *E. coli* F01 can be used as a microbial reactor for chondroitin biosynthesis (Figure 1d). However, the balance between the carbonization pool and the ammoniation pool still needs to be optimized to enhance the efficiency of the polymerization pool for chondroitin production.

### 2.2 Increasing the Capacity of the Ammoniation Pool

**Figure 2:**
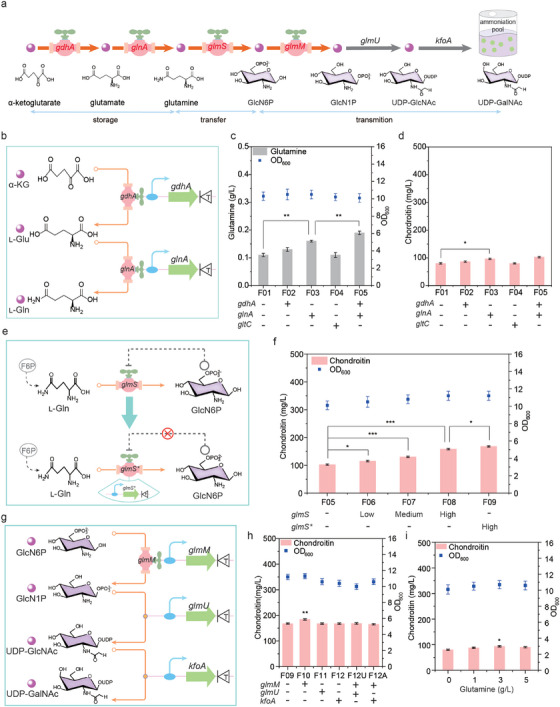
Increasing the capacity of the ammoniation pool for chondroitin production. a) The transformation strategy for increasing the ammoniation pool. GlcN‐6P, glucosamine‐6‐phosphate; GlcN‐1P, glucosamine‐1‐phosphate; UDP‐GlcNAc, UDP‐*N*‐acetylglucosamine; UDP‐GalNAc, UDP‐*N*‐acetylgalactosamine. b) Increasing the storage of ammonia in the ammoniation pool. α‐KG, α‐ketoglutarate; L‐Glu, glutamate; L‐Gln, glutamine. c) The effect of gene overexpression in glutamine pathway on the storage of ammonia. d) The effect of gene overexpression in glutamine pathway on chondroitin production. e) Improving the transfer of ammonia in the ammoniation pool. Aminotransferase (GlmS) catalyzed the conversion of F6P to GlcN6P. The efficiency of ammonia transfer was reduced by GlcN6P to inhibit the enzyme activity of GlmS. F6P, fructose‐6‐phosphate. f) The effect of GlmS expression on the transfer of ammonia. g) Enhancing the transmition of ammonia in the ammoniation pool. h) The effect of gene overexpression in UDP‐GalNAc pathway on chondroitin production. i) The effect of glutamine supplementation on chondroitin production. The data were presented as mean values ± SD from three independent biological replicates (n = 3). Statistical significance was evaluated using a two‐tailed Student's t‐test, and significance levels were denoted as ^*^ *p* <0.05, ^**^ *p* <0.01, ^***^ *p* <0.001.

The ammoniation pool was used for UDP‐GalNAc synthesis. To enlarge the capacity of the ammoniation pool, three strategies were carried out: increasing the storage of ammonia, improving the transfer of ammonia, and enhancing the transmition of ammonia (**Figure** **2**a).

To increase the storage of ammonia, we first analyzed the effect of glutamine on chondroitin production. When 1, 3, and 5 g L^−1^ glutamine was added into culture medium, chondroitin titer was increased to 88, 93.6, and 90.1 mg L^−1^, which was 9.9%, 16.9%, and 12.5% higher than that without glutamine addition, respectively (Figure 2i). The concentration of UDP‐GalNAc was increased to 0.19, 0.25, and 0.24 µm/gDCW, which was 58.3%, 108.3% and 100.0% higher than those of no glutamine addition, respectively (Figure S6, Supporting Information). These results suggested that increasing the supply of glutamine was favorable for chondroitin synthesis. Based on these observations, we next upregulated two ammonia flux valves, *gdhA* and *glnA* genes, to increase the supply of glutamine for the UDP‐GalNAc pathway, obtaining the recombinant strains *E. coli* F02 and *E. coli* F03, respectively. The concentration of glutamine showed a 18.2% and 45.5% increase up to 0.13 and 0.16 g L^−1^ (Figure 2b,c), and the titer of chondroitin was 86.5 and 96.6 mg L^−1^, which was increased by 8.0% and 20.6% compared with strain *E. coli* F01, respectively (Figure 2d). In addition, we also introduced a transcription regulator *bsgltC* derived from *Bacillus subtilis* to activate the biosynthetic pathway of glutamine, but the results showed that there was no effect on the accumulation of glutamine. Finally, to combinatorially enhance the supply of glutamine, ammonia flux valves, *gdhA* and *glnA* genes, were co‐upregulated, resulting in strain *E. coli* F05. The concentration of glutamine and UDP‐GalNAc was up to 0.19 g L^−1^ and 0.22 µm/gDCW, which was increased by 18.8% and 22.2% compared with strain *E. coli* F03, respectively (Figure 2c; Figure S7, Supporting Information). Meanwhile, the titer of chondroitin was up to 102.6 mg L^−1^, which was 6.2% higher than that of *E. coli* F03 (Figure 2d). These results indicated that the synergistic optimization of ammonia flux valves was beneficial for improving the efficiency of ammonia storage, thus enhancing chondroitin synthesis.

To improve the transfer of ammonia, ammonia flux valve, *glmS* gene, was upregulated. GlmS can transfer the amino group from glutamine to F6P to form GlcN‐6P, which was the core intermediate metabolites of ammoniation pool. To increase the accumulation of GlcN‐6P, ammonia flux valve, *glmS* gene, was regulated with three different strengths of RBS (RBSl: low strength, RBSm: medium strength, RBSh: high strength), obtaining strains *E. coli* F06, *E. coli* F07, and *E. coli* F08, respectively (Figure S4, Supporting Information). Chondroitin production with *E. coli* F06, *E. coli* F07, and *E. coli* F08 was up to 115.3, 130.5, and 158.2 mg L^−1^, which was increased by 12.4%, 27.2%, and 54.2% compared with strain *E. coli* F05, respectively (Figure 2f). These results indicated that regulating ammonia flux valve could enhance the transfer of ammonia, thus promoting chondroitin production. However, the accumulated GlcN‐6P could inhibit the catalytic efficiency of GlmS, thereby reducing ammonia transfer (Figure 2e). To relieve feedback inhibition, the mutanted ammonia flux valve, *glmS* ^*^ gene, was introduced into *E. coli* F08 to promote the transfer of ammonia, obtaining strain *E. coli* F09.^[^ ^15^ ^]^ Chondroitin production and UDP‐GalNAc accumulation of *E. coli* F09 reached 167.8 mg L^−1^ and 0.33 µm/gDCW, which was 6.1% and 10.0% higher than those of strain *E. coli* F08 (Figure 2f; Figure S8, Supporting Information). These results indicated that the mutanted ammonia flux valve can improve the transfer of ammonia by alleviating the inhibitory effect of GlcN‐6P, thereby enhancing chondroitin production.

To enhance the transmission of ammonia, ammonia flux valves, *glmM*, *glmU*, and *kfoA* genes, in the UDP‐GalNAc pathway was upregulated, respectively. We found that upregulation of ammonia flux valve, *glmM* gene, enhanced the production of chondroitin to 184.4 mg L^−1^, which was 9.9% higher than that of *E. coli* F09 (Figure 2g,h). However, upregulation of ammonia flux valves, *glmU* and *kfoA* genes, could not enhance chondroitin production. Further, combination upregulation of ammonia flux valves, *glmM*, *glmU*, and *kfoA* genes did not increase the production of chondroitin in *E. coli* F12U and F12A (Figure 2h). Thus, upregulation of key ammonia flux valve, *glmM* gene, was favorable for the transmission of ammonia, which was beneficial for chondroitin production.

To sum up, the capacity of the ammoniation pool could be enhanced to increase the production of chondroitin by increasing the storage of ammonia, improving the transfer of ammonia, and enhancing the transmission of ammonia.

### 2.3 Enhancing the Capacity of the Carbonization Pool

**Figure 3:**
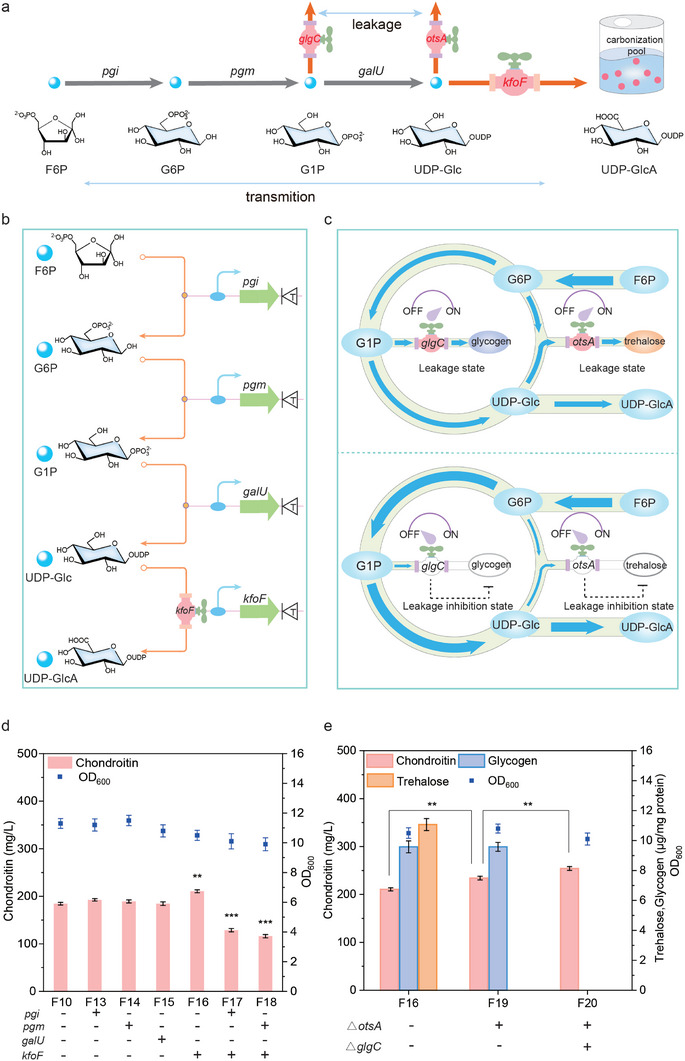
Enhancing the capacity of the carbonization pool for chondroitin production. a) The transformation strategy for increasing the carbonization pool. F6P, fructose‐6‐phosphate; G6P, glucose‐6‐phosphate; G1P, glucose‐1‐phosphate; UDP‐Glc, UDP‐glucose; UDP‐GlcA, UDP‐glucuronate; *glgC*, glucose‐1‐phosphate adenylyltransferase; *otsA*, trehalose‐6‐phosphate synthase. b) Enhancing the transmition of carbon in the carbonization pool. c) Blocking the leakage of carbon in the carbonization pool. Glucose‐1‐phosphate adenylyltransferase (GlgC) catalyzed the conversion of G1P to ADP‐Glc. Trehalose‐6‐phosphate synthase (OtsA) catalyzed the conversion of G6P and UDP‐Glc to trehalose‐6‐phosphate (T6P). d) The effect of upregulation of four carbon flux genes, *pgi*, *pgm*, *galU* and *kfoF*, in UDP‐GlcA pathway on transmition of carbon. e) Inhibiting the leakage of carbon by knocking out the biosynthesis of trehalose and Glycogen. Inhibition of the biosynthesis of trehalose precursor T6P by turning off *otsA*. Inhibition of the biosynthesis of glycogen precursor ADP‐Glc by turning off *glgC*. The data were presented as mean values ± SD from three independent biological replicates (n = 3). Statistical significance was evaluated using a two‐tailed Student's t‐test, and significance levels were denoted as ^*^ *p* <0.05, ^**^ *p* <0.01, ^***^ *p* <0.001.

The carbonization pool was used for UDP‐GlcA synthesis. To expand the capacity of the carbonization pool, two strategies were adopted: enhancing the transmission of carbon and blocking the leakage of carbon (**Figure** **3**a).

To enhance the transmission of carbon in the carbonization pool, four carbon flux valves, *pgi*, *pgm*, *galU* and *kfoF* genes, were upregulated in UDP‐GlcA pathway (Figure 3b). We found that the upregulation of *pgi*, *pgm*, and *kfoF* increased chondroitin production to 192.4, 189.2, and 210.6 mg L^−1^, which was 4.3%, 2.6%, and 14.2% higher than those of strain *E. coli* F10, respectively (Figure 3d), but the upregulation of *galU* had no impact on chondroitin synthesis. Further, when *kfoF* and *pgi* (or *pgm*) were upregulated simultaneously, chondroitin production was decreased by 30.2% (or 37.1%). Therefore, carbon flux valve, *kfoF* gene, was essential for the transmission of carbon.

To block the leakage of carbon in the carbonization pool, we turned off the key carbon flux valves of trehalose and glycogen synthesis (Figure 3c). First, trehalose synthesis required G6P and UDP‐Glc as precursors, which would lead to the leakage of carbon for UDP‐GlcA synthesis.^[^ ^16^ ^]^ To inhibit this carbon leakage from the carbonization pool to trehalose, we turned off the key carbon flux valve, *otsA* gene, in *E. coli* F16, which could catalyze the synthesis of trehalose‐6‐phosphate, obtaining strain *E. coli* F19. As we speculated, trehalose was not detected, but there was no effect on cell growth. Meanwhile, the production of chondroitin reached 234.2 mg L^−1^, which was 11.2% higher than that of *E. coli* F16 (Figure 3e). Second, *E. coli* could accumulate a certain amount of glycogen by consuming G1P,^[^ ^17^ ^]^ which would exacerbate the leakage of carbon in carbonization pool. To inhibit this carbon leakage from the carbonization pool to glycogen, we turned off the key carbon flux valve, *glgC* gene, in *E. coli* F19, obtaining strain *E. coli* F20. Based on this, the accumulation of glycogen was not detected. Although the OD_600_ of the strain *E. coli* F20 was decreased by 8%, chondroitin production reached 254.3 mg L^−1^, which was 8.6% higher than that of strain *E. coli* F19 (Figure 3e). These results indicated that blocking the synthesis of trehalose and glycogen could inhibit the leakage of carbon, leading to a large increase in chondroitin production.

To sum up, enhancing the transmission of carbon and blocking the leakage of carbon were beneficial for improving the capacity of the carbonization pool for increasing chondroitin synthesis.

### 2.4 Balancing the Ammoniation and Carbonization Pools Using Molecular Valves

#### 2.4.1 Designing Molecular Valves to Regulate Gene Expression

**Figure 4:**
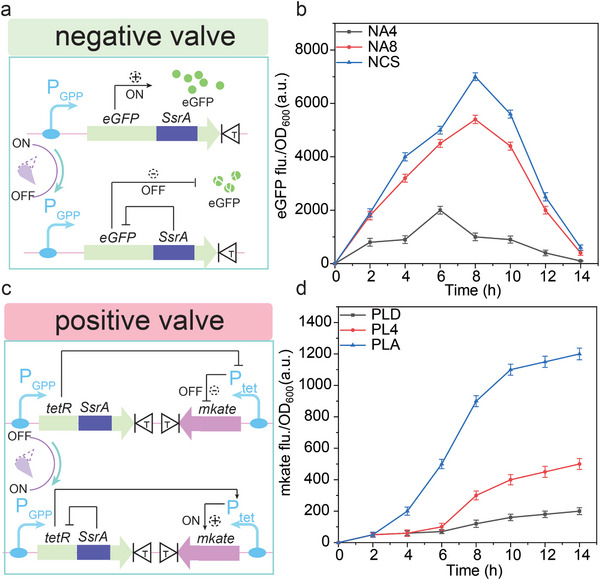
Designing molecular valves to regulate gene expression. a) Design and characterization of negative valve. PGPP, growth phase‐dependent promoter. SsrA, C‐terminal degradation sequence; T, T7 terminator. b) The fluorescence abundence of different negative valves. c) Design and characterization of positive valve. d) The fluorescence abundence of different positive valves. The values and error bars reflected the mean ± standard deviations (SD) of three biological replicates (n = 3).

The synthesis of chondroitin was involved to a synergistic effect of the ammoniation and carbonization pools. Thus, the growth phase promoter and degradation tag were used to construct a bidirectional molecular valve to balance the ammoniation and carbonization pools.^[^ ^18^ ^]^ This molecular valve was composed of a positive valve and a negative valve. As shown in **Figure** **4**a, the negative valve was constructed to regulate target genes that need to be closed by combining a growth stage promoter and degradation tag. Specifically, the target genes constitutively degraded based on C‐terminal signals could be rapidly synthesized during the growth phase, whereas terminated when the cells reached the stationary phase. The positive valve was constructed to regulate target genes that need to be opened by combining a growth phase promoter and a repressor activated by an inducible promoter (Figure 4c). To construct this positive valve, the targeted gene was modified to be inhibited by fusing a degradation tag in the C terminal. Then, the TetR repressor was modified by fusing a degradation tag in the C terminal. With the degradation of TetR repressor, the targeted gene controlled by inducible Ptet promoter would be upregulated. To characterize the behavior of negative valve in *E. coli* BL21 STAR, three molecular valves with different regulation levels were introduced.^[^ ^19^ ^]^ As the reporter of negative valve, eGFP was controlled by PGPP promoter and fused with a degradation tag in the C terminal (Figure 4a). Based on this, we constructed three negative valves, Prrnc‐egfp‐DAS (NCS), PrrnA‐egfp‐DAS+8 (NA8) and PrpsA‐egfp‐DAS+4 (NA4), by combining growth stage promoters and degradation tags. To characterize the regulatory ability of these negative valves, the relative fluorescence value of eGFP in strains was determined. The fluorescence value was all first increased and then decreased, and further analysis showed that the inhibitory ability of negative valves NCS, NA8 and NA4, were increased orderly (Figure 4b). To characterize the behavior of positive valve, three molecular valves with different regulatory levels were introduced. As the reporter of positive valves, mKate was controlled by Ptet promoter, and Ptet promoter was activated when the TetR repressor was degradated (Figure 4c). Based on this, three positive valves, PrpsL‐tetR‐LAA‐Ptet‐mKate (PLA), PrpsL‐tetR‐DAS+4‐Ptet‐mKate (PL4) and PrpsL‐tetR‐GSD‐Ptet‐mKate (PLD) were constructed by combining a growth phase promoter and a repressor activated by an inducible promoter. To characterize the regulatory ability of three positive valves, the relative fluorescence value of *mkate* in strains was measured. The fluorescence value was all inhibited during the growth phase and activated until the stationary phase. Further analysis showed that the activation ability of positive valves PLD, PL4, and PLA were increased orderly (Figure 4d).

#### 2.4.2 Weakening the Competitive Pathways Using Molecular Valves

**Figure 5:**
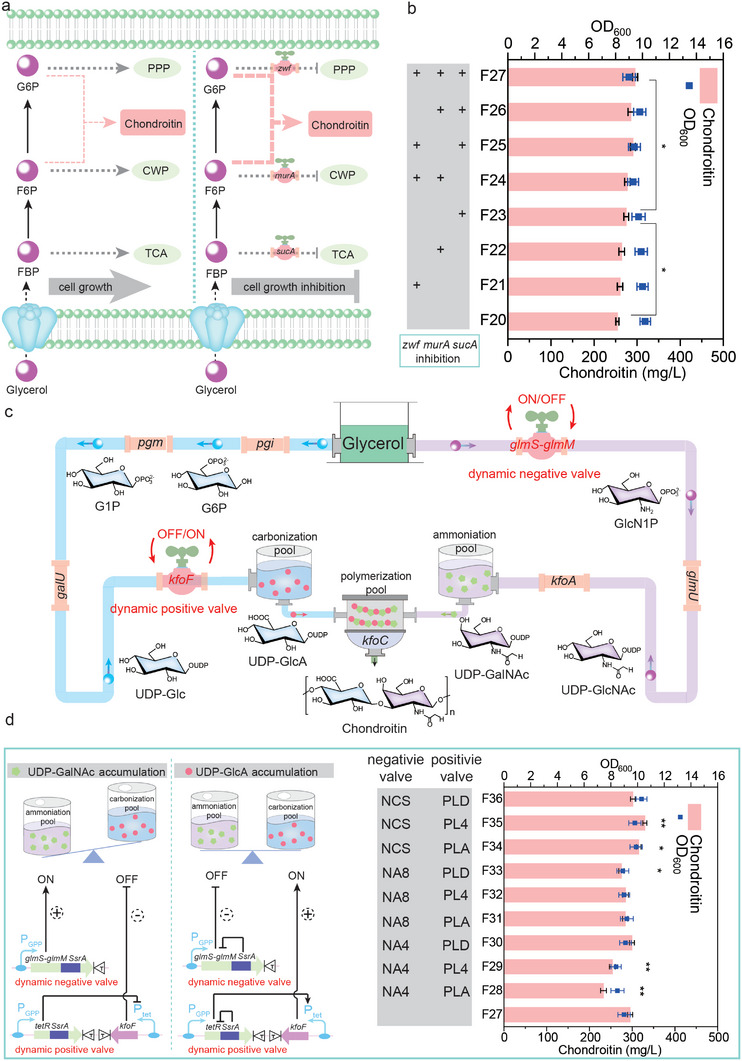
Balancing ammoniation pool and carbonization pool using molecular valves. a) The main competitive pathways in *E. coli* and the pathways under the regulation of molecular valves in *E. coli*. PPP, pentose phosphate pathway; CWP, cell wall synthesis pathway; TCA, citric acid cycle pathway; *zwf*, glucose‐6‐phosphate 1‐dehydrogenase; *murA*, UDP‐*N*‐acetylglucosamine 1‐carboxyvinyltransferase; *sucA*, 2‐oxoglutarate dehydrogenase E1 component. b) The effect of inhabiting the main competitive pathways on chondroitin production. c) Engineering the biosynthetic pathway of chondroitin in *E. coli* using molecular valves. d) Balancing ammoniation pool and carbonization pool using molecular valves in *E. coli*. KfoF was controlled by a positive valve to regulate carbonization pool. *GlmS*‐*glmM* was controlled by a negative valve to regulate ammoniation pool. The data were presented as mean values ± SD from three independent biological replicates (n = 3). Statistical significance was evaluated using a two‐tailed Student's t‐test, and significance levels were denoted as ^*^ *p* <0.05, ^**^ *p* <0.01, ^***^ *p* <0.001.

As shown in **Figure** **5**a, the citric acid cycle pathway (TCA), pentose phosphate pathway (PPP), and cell wall synthesis pathway (CWP) are the main competitive pathways, which would compete carbon source for chondroitin synthesis.^[^ ^20^ ^]^ Therefore, it was necessary to redistribute carbon flux from competitive pathways to the synthesis of chondroitin. However, these three pathways were related to cell growth. We speculated that knocking out key pathway enzymes in these competitive pathways may affect cell growth.^[^ ^21^ ^]^ Based on this, the dynamic regulation strategy directed a large amount of carbon flux toward the synthesis of products while meeting the basic growth conditions of strains. Here, the molecular valve strategy was applied to regulate the distribution of carbon flux, which could simultaneously weaken the competitive pathways and activate the chondroitin synthesis pathway. Glucose‐6‐phosphate 1‐dehydrogenase (*zwf*), UDP‐*N*‐acetylglucosamine 1‐carboxyvinyltransferase (*murA*) and 2‐oxoglutarate dehydrogenase E1 component (*sucA*) are key enzymes for connecting chondroitin synthesis with PPP, CWP and TCA, respectively. By down‐regulating *zwf* expression, weakening *murA* expression and inhibiting *sucA* expression, the accumulation of hyaluronic acid, UDP‐GlcNAc and 5 aminolevulinic acid were increased, respectively.^[^ ^22^ ^]^ Thus, to weaken chondroitin competitive pathways, *zwf*, *murA*, and *sucA* genes in PPP, CWP, and TCA modules were selected as regulatory targets with negative valve, respectively (Figure 5a). By combining competitive pathways, a total of seven recombinant strains were obtained to reduce the competitive consumption of carbon flux and promote chondroitin synthesis. As shown in Figure 5b, the individual inhibition of *zwf*, *murA*, and *sucA* expression could inhibit cell growth, but all inhibition increased chondroitin synthesis. Among them, when *sucA* gene was inhibited in strain *E. coli* F23, chondroitin production reached 274.6 mg L^−1^, which was 8.0% higher than that of strain *E. coli* F20, but OD_600_ showed a 5.2% reduction. By further weakening *zwf*, *murA*, and *sucA* genes of competitive pathways simultaneously, the resulting strain *E. coli* F27 showed a 7.5% increase in chondroitin production up to 295.2 mg L^−1^, but OD_600_ was reduced by 7.2% compared with that of strain *E. coli* F23. These results indicated that inhibiting the distribution of carbon flux in competitive pathways could redirect more carbon flux toward the pathway of chondroitin synthesis, thereby increasing the production of chondroitin.

#### 2.4.3 Rematching the Ammoniation and Carbonization Pools Using Molecular Valves

Insufficient and imbalanced metabolic flux were key factors for affecting the efficiency of the polymerization pool. On the basis of increasing the storage of ammoniation and carbonization pools, the balance of metabolic flux was a crucial strategy to further enhance the production of chondroitin. Based on the above‐mentioned results, we have identified *kfoF*, *glmS*‐*glmM* as key valves for regulation in the ammoniation and carbonization pools. Here, the bidirectional molecular valve strategy was applied to regulate the balance between the ammoniation and carbonization pools (Figure 5c). To balance the ammoniation pool and the carbonization pool, *glmS*‐*glmM* were selected as regulatory targets with negative valve, because *glmS*‐*glmM* were used not only for the supply of chondroitin precursors in the ammoniation pool, but also for cell growth in the CWP. *kfoF* was selected as regulatory target with positive valve, because *kfoF* was used for the supply of chondroitin precursors in the carbonization pool (Figure 5d). Thus, the carbonization and ammoniation pools were optimized to enhance chondroitin synthesis by combinatorially using negative and positive valves, obtaining a total of nine recombinant strains. As shown in Figure 5d, the titer of chondroitin was ranged from 232.8 to 329.6 mg L^−1^ among recombinant strains. By comparing chondroitin synthesis in different strains, strain *E. coli* F35 produced chondroitin up to 329.6 mg L^−1^, which was 11.7% higher than that of strain *E. coli* F27, and its OD_600_ was increased by 8.9%. Thus, *E. coli* F35 was selected for subsequent experiments. These results suggested that carbon flux between the ammoniation and carbonization pools was redirected into chondroitin synthesis pathway by using the bidirectional molecular valves.

### 2.5 Reprogramming the Adaptability of Polymerization Pool with the Ammoniation and Carbonization Pools

**Figure 6:**
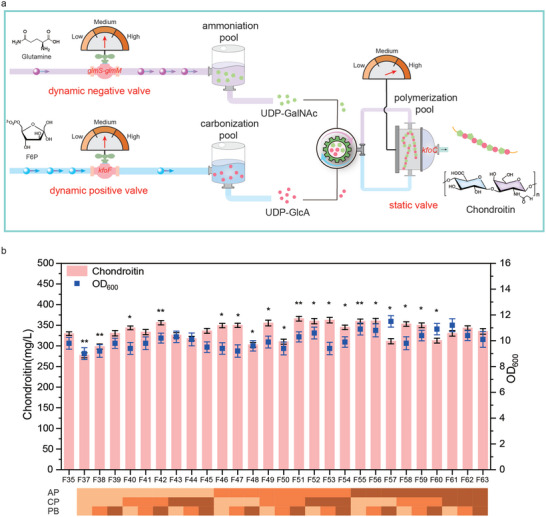
Reprogramming the adaptability of the polymerization pool. a) Designing different regulation combinations among polymerization pool, ammoniation pool, and carbonization pool with different adapters. b) The effect of different adapters on chondroitin production. AP, the ammoniation pool; CP, the carbonization pool; PB, the polymerization pool. The data were presented as mean values ± SD from three independent biological replicates (n = 3). Statistical significance was evaluated using a two‐tailed Student's t‐test, and significance levels were denoted as ^*^ *p* <0.05, ^**^ *p* <0.01, ^***^ *p* <0.001.

In the process of chondroitin synthesis, the ammoniation and carbonization pools were directed toward the polymerization pool for chondroitin synthesis (**Figure** **6**a). In addition to the flux balance between the ammoniation and carbonization pools, the adaptability of the polymerization pool with the ammoniation and carbonization pools was also important for chondroitin synthesis. To improve the catalytic efficiency of the polymerization pool, chondroitin production was effectively increased by upregulating the polymerization valve, *kfoC* gene.^[^ ^23^ ^]^ However, the excessive upregulation of *kfoC* would actually reduce the synthesis of chondroitin.^[^ ^9^ ^]^ Therefore, improving the catalytic efficiency of the polymerization pool was an effective strategy to increase carbon flux from the ammoniation and carbonization pools to the polymerization pool.

To improve the adaptability of polymerization pool with the ammoniation and carbonization pools, the metabolic flux among polymerization pool, ammoniation pool, and carbonization pool modules was rematched by improving the transfer efficiency of metabolic flux. Here, three types of adapters were developed by reinstalling dynamic positive valve, dynamic negative valve, and static valve with different strength RBSs to fine‐tune the adaptation combinations among three modules. Specifically, these adapters were applied to control the key pathway genes, *kfoC* gene in the polymerization pool with static valve, *glmS*‐*glmM* genes in the ammoniation pool with dynamic negative valve and *kfoF* gene in the carbonization pool with dynamic positive valve, forming 27 adapter combinations. After that, we evaluated the effect of these combinations on chondroitin production. As shown in Figure 6b, the production of chondroitin was ranged from 277.1 to 365.7 mg L^−1^, while the final OD_600_ was ranged from 9.0 to 11.5, indicating that different adapter combinations significantly affected cell growth and the efficiency of chondroitin synthesis. By comparing 27 recombinant strains, we found strain *E. coli* F51 showed a 11.0% improvement in chondroitin production up to 365.7 mg L^−1^ and a slight improvement in final OD_600_ up to 10.3. However, the other adapters, too strong or too weak, reduced the adaptability among polymerization pool, the carbonization pool, and the ammoniation pool, which was not favored for chondroitin synthesis. These results indicated that adjusting the strength of adapters to promote the adaptability among polymerization pool, carbonization pool, and ammoniation pool, thus increasing the production of chondroitin.

### 2.6 Scale‐Up Production for Chondroitin in a 5‐L Bioreactor

**Figure 7:**
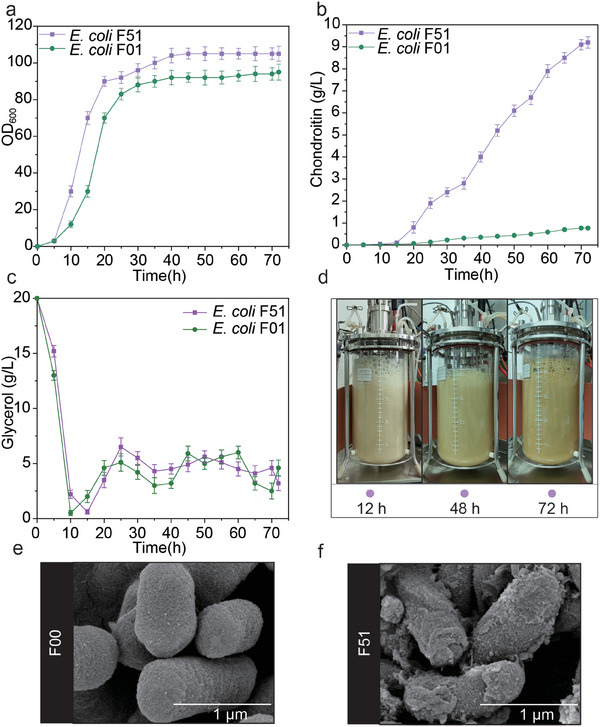
Scale‐up production for chondroitin in a 5‐L bioreactor. a) Cell growth of strain *E. coli* F01 and F51. b) Chondroitin production of strain *E. coli* F01 and F51. c) Glycerol feeding of strain *E. coli* F01 and F51. d) Fermentation process of strain *E. coli* F51. (e) and (f) Morphological of *E. coli* F00 and F51. The values and error bars reflected the mean ± standard deviations (SD) of three biological replicates (n = 3).

The optimal strain *E. coli* F51 was tested in a 5‐L bioreactor with fed‐batch fermentation for chondroitin production. As shown in **Figure** **7**a,d, *E. coli* F51 continued to grow from 0 to 40 h, with biomass peaking at 45 h. Further, chondroitin continued to accumulate during the whole process of fermentation. The maximum OD_600_ and chondroitin production reached 105 and 9.2 g L^−1^, which was 10.5% and 1094.8% higher than that of *E. coli* F01, respectively (Figure 7a–c; Figure S5, Supporting Information). In addition, chondroitin, as a polysaccharide polymer, had a certain viscosity and tended to adhere to the surface of host strain. To further verify the accumulation of chondroitin, scanning electron microscope was employed to detect differences on the surfaces of *E. coli* F00 and *E. coli* F51.We found a large amount of chondroitin on the surface of strain *E. coli* F51, but there was no accumulation on the surface of strain *E. coli* F00 (Figure 7e,f). The *M*_w_ and *M*_n_ of chondroitin produced by strain *E. coli* F51 reached 2289.2 and 912.5 kDa, indicating that the generated chondroitin was the high‐molecular‐weight polysaccharide (**Table** **1**). These results showed that the recombination strain *E. coli* F51 was favorable for biosynthesizing chondroitin.

**Table 1:** Molecular weight of chondroitin.

Strains	*M*_w_[kDa]^a)^	*M*_n_[kDa]^b^ ^)^	*I*p^c^ ^)^
*E. coli* F00	‐	‐	‐
*E. coli* F51	2289.2	912.5	2.5

^a)^ weight average molecular weight;

^b)^ number average molecular weight;

^c)^ polydispersity index (*M*_w_/*M*_n_).

## 3 Discussion

Flux size refers to transmission throughput of metabolic flux for converting substrates into products in MCFs, essentially for transmitting in the biosynthetic pathways of targeted chemicals. Regulating flux size has been widely used in improving pathway efficiency for bioproduction of chemicals, such as natural pathways of endogenous products,^[^ ^24^ ^]^ reconstructed pathways of heterologous products,^[^ ^25^ ^]^ and artificially designed pathways of new‐to‐nature products.^[^ ^26^ ^]^ Three strategies have been used for the regulation of flux size, such as enhancing the supply of precursors,^[^ ^8^ ^]^ shutting down the decomposition of intermediates,^[^ ^27^ ^]^ and deregulating the feedback inhibition of products.^[^ ^28^ ^]^ Chondroitin is the amino‐containing polysaccharide, so the coordination between carbon and nitrogen flux is crucial for the biosynthesis of chondroitin. In these strategies, they just take carbon flux into account, not focus on the coordination between carbon flux and nitrogen flux. For example, heparosan backbone mainly consists of carbon and nitrogen. Heparosan production was increased to 1.29 g L^−1^ by regulating carbon flux to enhance the supply of precursors with co‐expression of *bs*GalU (glucose‐1‐phosphate uridylyltransferase), *ec*KfiD (UDP‐glucose‐6‐dehydrogenase), ecGlmM (phosphoglucosamine mutase) and *ec*KfiAC (GlcNAc transferase and GlcA transferase).^[^ ^29^ ^]^ Although this carbon flux size regulation can enlarge the capacity of carbon pool to a certain extent for improving the biosynthesis of chemicals, it cannot systematically regulate flux size in complex metabolic pathways from one‐pronged approach. In our study, chondroitin backbone is mainly formed by carbon and nitrogen. From this angle embarking, we constructed the ammoniation pool for UDP‐GalNAc synthesis and the carbonization pool for UDP‐GlcA synthesis, which was convenient for systematically regulating transmission throughput of metabolic flux to produce chondroitin in a microbial reactor.

Flux direction is transmission trajectory of metabolic flux toward targeted products, which is usually used to orderly guide the directed transmission of metabolic flux. Flux direction has become an effective strategy for increasing pathway efficiency of chemicals, including growth‐dependent products, partially growth‐dependent products, and non‐growth‐dependent products.^[^ ^30^ ^]^ Three strategies have been applied to regulate flux direction, such as decoupling cell growth and product synthesis, inhibiting by‐product synthesis, and cutting off the competitive branching pathways.^[^ ^22^, ^31^ ^]^ These strategies have achieved to shift flux direction from the competitive pathways to product synthesis pathways, but they cannot reasonably redistribute the metabolic flux derived from the competitive pathways for product synthesis, due to the fact that long‐chain molecules usually consist of multiple precursor pools. For example, hyaluronic acid contains two precursor pools. To direct transmission of metabolic flux for producing hyaluronic acid, *pfkA* and *zwf* related to glycolysis and pentose phosphate pathway were co‐repressed based on CRISPR interference.^[^ ^22a^ ^]^ The resulting strain *B. subtilis* AW019‐3 realized a significant increase in hyaluronic acid production up to 2.26 g L^−1^, which was 108% higher than that of control strain *B. subtilis* AW009. Although the flux direction transfer from cell growth to hyaluronic acid pathway enhances the supply of metabolic flux for hyaluronic acid, it is not possible to rationally balance the distribution of metabolic flux among different precursor pools. In our study, we achieved the rational distribution of metabolic flux between the ammoniation pool and the carbonization pool using molecular valves, which could shift metabolic flux from cell growth to chondroitin synthesis.

Flux rate is transmission speed of metabolic flux from substrates to products, which is able to alter the transmission process of metabolic flux in the temporal dimension. Flux rate has become a useful strategy for enhancing pathway efficiency of metabolic flux in simple metabolic pathways, long metabolic pathways, and complex metabolic pathways.^[^ ^32^ ^]^ Three strategies have been explored to regulate flux rate, such as constructing fusion proteins, synthetizing biological scaffolds, and building spatial compartmentalization.^[^ ^9^, ^33^ ^]^ In these strategies, they locally increased the transmission speed of metabolic flux in the constructed metabolite channel. However, they overlooked the speed of substrates accessed into channel through free diffusion, which inhibited flux rate for bioproduction. Recently, based on the specific interaction of zinc finger proteins, GlmS and GNA1 was targeted and binded to synthetic DNA sequences for regulating the ratio of pathway enzymes.^[^ ^33a^ ^]^ In strain *B. subtilis* BSGN4‐S2, the titer of GlcNAc (4.5 g L^−1^) was increased by 2.5‐fold compared with that of strain *B. subtilis* BSGN4. Although metabolite channel promotes the transmission speed of intermediates for product synthesis, it still cannot prevent the escape of intermediates in the process of precursor supply for product synthesis in complex metabolic pathways. In our study, a centralization‐to‐transmission strategy was used to first centralize metabolic flux from substrates to precursors (UDP‐GalNAc and UDP‐GlcA), and then precursors were transmitted for product synthesis (chondroitin). Based on the ammoniation and carbonization pools, we used adapters to rematch the adaptability of polymerization pool with precursor pools, which enhanced flux rate for chondroitin synthesis.

Taken together, reprogramming metabolic flux provides a platform to build MCFs for improving the synthesis efficiency of bioproduction. This method not only systematically optimizes transmission of metabolic flux, but also visually simplifies the complex metabolic networks. Compared to previous studies based on static tools, dynamic valves were designed and constructed in our study to systematically regulate the redistribution of metabolic flux for promoting chondroitin production. As a result, chondroitin production with the engineered *E. coli* F51 reached 9.2 g L^−1^, which was the highest reported production (Table S4, Supporting Information). Further, this method can to some extent realize the division of labor of metabolic flux, thus increasing the efficiency of MCFs.

## 4 Conclusion

In this study, metabolic flux was systematically reprogrammed by regulating flux size, flux direction and flux rate to build an efficient MCF for chondroitin bioproduction. First, flux size was remade to improve transmission throughput of metabolic flux by enlarging the capacity of the carbonization and ammoniation pools. Then, flux direction was redistributed to direct transmission trajectory of metabolic flux by rematching the carbonation and ammoniation pools using molecular valves. Further, flux rate was fine‐tuned to control transmission speed of metabolic flux by optimizing the adaptability of polymerization pool with the ammoniation and carbonization pools. Finally, the engineered strain *E. coli* F51 produced 9.2 g L^−1^ chondroitin in 5‐L bioreactor. This strategy shown here provides a systematical approach for regulating metabolic flux in complex metabolic pathways for efficient biosynthesis of chemicals.

## 5 Experimental Section

### Strains and Plasmids

The strain *E. coli* BL21 STAR (DE3) was purchased from Novagen and was employed for chondroitin production. Strains used in this study were listed in Table S1 (Supporting Information). *E. coli* JM109 was utilized for the construction of recombinant plasmids. All plasmids used in this study are listed in Table S2 (Supporting Information).

### Culture Medium

A defined medium was employed as fermentation medium.^[^ ^9^ ^]^ A defined medium (1 L) contained 20 g L^−1^ glycerol, 13.5 g L^−1^ KH_2_PO_4_, 4 g L^−1^ (NH_4_)_2_HPO_4_, 1.7 g L^−1^ citric acid, 1.4 g L^−1^ MgSO_4_·7H_2_O, 4.5 mg L^−1^ vitamin B1, and 10 mL L^−1^ trace metal solution, pH 7.0. The trace metal solution dissolved with 0.1 mm HCl, including 10.0 g L^−1^ FeSO_4_·7H_2_O, 0.5 g L^−1^ MnSO_4_·4H_2_O, 2.2 g L^−1^ ZnSO_4_·7H_2_O, 1.0 g L^−1^ CuSO_4_·5H_2_O, 2.0 g L^−1^ CaCl_2_, 0.02 g L^−1^ Na_2_B_4_O_7_·10H_2_O, 0.1 g L^−1^ (NH_4_)_6_Mo_7_O_24_·4H_2_O. This trace metal solution was also added to fermentation medium. In 5‐L fermenter, the component of feeding solution contained 500 g L^−1^ glycerol, 10 g L^−1^ MgSO_4_·7H_2_O, and 0.2 g L^−1^ vitamin B1. Appropriate antibiotics, contained Ampicillin (100 mg L^−1^), kanamycin (50 mg L^−1^), or streptomycin (30 mg L^−1^), were added as needed.

### Fermentation Conditions

For chondroitin production in shaker flasks, the engineered *E. coli* strain was initially cultured on agar plates overnight at 37 °C. Subsequently, to establish seed culture, a single colony was grown in 30 mL of LB medium for 12 h. Finally, seed culture was inoculated with 500 µL into 50 mL of fermentation medium and cultivated at 37 °C for 72 h, 200 rpm. When the growth of engineered strains (OD_600_) reached 0.8, the corresponding inducer (IPTG) was added. For chondroitin production in a 5‐L fermenter (Intelli‐Ferm A‐5L, Parallel‐Bioreactor, Shanghai, China), seed cultures were prepared by similar methods. Next, 10% (vol/vol) of seed culture was inoculated into 2 L of fermentation medium. The engineered strains were induced with 0.4 mm IPTG when OD_600_ reached 10. Throughout culture process, pH was automatically monitored and maintained at 7.0 by adding ammonium hydroxide as needed. Upon consumption of the supply of glycerol in fermentation medium, glycerol was fed to maintain glycerol concentration at 5 g L^−1^.

### DNA Manipulation

Gibson assembly and molecular cloning techniques were employed for plasmid construction. The genes *kfoC*, *kfoF* and *kfoA*, essential for constructing the chondroitin biosynthesis pathway, were PCR‐amplified from the genomic DNA of *E. coli* K4. In addition, the genes *glnA*, *gdhA*, *glmS*, *glmM*, *glmU*, *galU*, *pgm* and *pgi* were PCR‐amplified from the genomic DNA of *E. coli* BL21 STAR (DE3). The gene GlmS* was synthesized by talen‐bio (Wuxi, China).^[^ ^15^ ^]^ CRISPR‐Cas9 was employed to delete target genes in the genome of engineered *E. coli*.^[^ ^34^ ^]^ To knock out *glgC* and *otsA* genes, the corresponding single‐guide RNA (sgRNA) sequences in plasmid pTargetF and donor DNA were designed. These constructs were transformed into engineered strains with pCas9. Finally, single colonies that survived on agar plates were further characterized and identified.

### Scanning Electron Microscopy

Fermentation samples of 10 mL were collected from fermenters and centrifuged at 4000 rpm for 2 min. The precipitated samples were washed with PBS (pH 7.0) and centrifuged at 4000 rpm for 2 min. Finally, the precipitated samples were fixed with 2.5% glutaraldehyde solution and stored at 4 °C for 12 h. The prepared samples were subsequently analyzed using scanning electron microscopy at Shiyanjia Lab (Hangzhou, China).

### Staining of Chondroitin

First, the engineered *E. coli* was cultured for 72 h and diluted appropriately for application onto a microscope slide. Next, the sample was allowed to air‐dry at room temperature. Subsequently, crystal violet dye was employed to stain the dried sample for 5 min. Finally, the stained sample was washed three times with 20% copper sulfate solution and subjected to observation using an optical microscope.^[^ ^35^ ^]^

### Analytical Methods

The OD_600_ value was monitored by a spectrophotometer. The concentration of glycerol was determined using the M‐100 Biosensors Analyzer (Shenzhen Sieman Technology Co., Ltd). L‐glutamine was analyzed using the UV detector of an Agilent 1260, and the absorbance was measured at 254 nm following derivatization with phenyl isothiocyanate.^[^ ^36^ ^]^ UDP‐GalNAc was detected by capillary electrophoresis on HPCE (P/ACE MDQ; Beckman Coulter) with a UV lamp and a diode array detector, using an uncoated fused‐silica capillary (50 µm internal diameter, 70 cm total length, 60 cm effective length; Beckman Coulter).^[^ ^9^ ^]^ Intracellular glycogen and trehalose concentrations were quantified utilizing specific assay kits. The trehalose content was assessed with a trehalose content assay kit, and the glycogen content was determined using a glycogen content assay kit. These assay kits were procured from Sangon Biotech (Shanghai, China).

To measure chondroitin, the fermentation broth was centrifuged at 12 000 rpm for 10 min to collect the supernatant. This supernatant was mixed with three volumes of ethanol, and chondroitin was precipitated at 4 °C for 8 h. Subsequently, the mixture was centrifuged at 12 000 rpm for 10 min to collect chondroitin, which was dried completely at 65 °C. Chondroitin was dissolved in 1 mL deionized water, and these steps were repeated more than three times. A carbazole assay was employed to determine chondroitin concentration.^[^ ^9^ ^]^ Chondroitin was lyophilized and redissolved in 20 mm Tris‐HCl buffer (pH 7.4). Next, 10 U mL^−1^ chondroitinase ABC was added and maintained at 37 °C for 24 h. Finally, reaction mixture was filtered through a 0.22 µm membrane. High‐performance liquid chromatography (HPLC) was applied to analyze the disaccharide units in conjunction with electrospray ionization mass spectrometry (ESI‐MS) in negative scan mode. The analysis was conducted using WATERS QUATTRO PREMIER XE, with a CSHC18 column (2.1 × 100 mm, 1.7 µm). The mobile phases consisted of 10 mm ammonium acetate (Phase A) and acetonitrile (Phase B), respectively. The column temperature was maintained at 40 °C, and elution proceeded at a linear gradient of 0.2 mL min^−1^ for 45 min.

### Sulfation of Chondroitin

To further confirm the biosynthesis of chondroitin, the purified enzymes (KlATPS, PcAPSK, and HsCHST11) were incubated with 60 mm ATP, 100 mm Na_2_SO_4_, and 10 g L^−1^ chondroitin to generate CSA.^[^ ^14^ ^]^ The lysis and product identification methods of CSA were conducted similarly to the method used for measuring chondroitin.

### Measurement of Chondroitin Weight Average Molecular Weight

Chondroitin was dissolved in ultrapure water and filtered through 0.22 µm membrane. High‐performance liquid chromatography‐exclusion chromatography (HPLC‐SEC) was employed, using an Ultrahyfrogel column (300 mm × 7.8 mm i.d., Waters Corporation, Milford, MA, USA) combined with a refractive index detector to measure the molecular weight of chondroitin. The mobile phase consisted of 0.1 m NaNO_3_ and the flow rate was maintained at 0.9 mL min^−1^.

### Assay of Fluorescence Intensity

The recombinant *E. coli* strain was first cultured on agar plates overnight at 37 °C. Subsequently, to establish seed culture, a single colony was cultivated in 30 mL of LB medium for 12 h. Once again, seed culture was inoculated with 500 µL into 50 mL of LB medium and grown at 37 °C, 200 rpm. Finally, the fluorescence intensity of fermentation broth was determined using an enzyme‐linked immunosorbent assay. To measure the fluorescence intensity of GFP, the excitation and emission wavelengths were monitored at 488 ± 10 and 512 ± 10 nm, respectively. For measuring the fluorescence intensity of mkate, the excitation and emission wavelengths were monitored at 588 ± 10 and 645 ± 10 nm, respectively.

### Statistical Analysis

All experiments were independently conducted at least three times, and the data were presented as mean values ± standard deviation (SD). Statistical data analysis was performed using the two‐tailed Student's t‐test in Excel (Microsoft Excel 2016). *P* values were represented as ^*^ *p* <0.05, ^**^ *p* <0.01, or ^***^ *p* <0.001, which were considered significant.

## Conflict of Interest

The authors declare no conflict of interest.

## Author Contributions

C.Z. conceived the study and made contributions to the design of the experiments, the acquisition of data, the analysis, and interpretation of data, and contributed to the manuscript writing. X.C. made contributions to the design of the experiments, the acquisition of data. X.L., L.G., C.G., W.S., W.W., J.W., and L.L. conceived and organized the study and helped to draft the manuscript, and have revised the manuscript. All authors read and approved the final manuscript.

## Supporting information

Supporting Information

## Data Availability

The data that support the findings of this study are available in the supplementary material of this article.
