# Supplementary material for: Reprogramming Metabolic Flux in Escherichia Coli to Enhance Chondroitin Production
Source: Adv Sci (Weinh). 2023 Dec 25;11(10):2307351. doi: 10.1002/advs.202307351 (PMC10933623; doi:10.1002/advs.202307351)
Supplement: Supplementary file 1 — Supporting Information [file ADVS-11-2307351-s001.pdf]

ADVANCED SCIENCE
Open Access

Supporting Information

for *Adv. Sci.*, DOI 10.1002/advs.202307351

Reprogramming Metabolic Flux in *Escherichia Coli* to Enhance Chondroitin Production

*Chunlei Zhao, Xiaomin Li, Liang Guo, Cong Gao, Wei Song, Wanqing Wei, Jing Wu, Liming Liu and Xiulai Chen**

Supporting Information, ©Wiley-VCH 2021, 69451 Weinheim, Germany

Reprogramming Metabolic Flux in *Escherichia coli* to Enhance Chondroitin Production

Chunlei Zhao,[a,b] Xiaomin Li,[a,b] Liang Guo,[a,b] Cong Gao,[a,b] Wei Song,[c] Wanqing Wei,[a,b] Jing Wu,[c] Liming Liu,[a,b] Xiulai Chen*[a,b]

[a] Dr. C. Zhao, Dr. X. Li, Dr. L. Guo, Dr. C. Gao, Dr. W. Wei, Prof. L. Liu, Prof. X. Chen
State Key Laboratory of Food Science and Resources
Jiangnan University
Wuxi 214122, China
E-mail: xlchen@jiangnan.edu.cn

[b] Dr. C. Zhao, Dr. X. Li, Dr. L. Guo, Dr. C. Gao, Dr. W. Wei, Prof. L. Liu, Prof. X. Chen
International Joint Laboratory on Food Safety
Jiangnan University
Wuxi 214122, China

[c] Dr. W. Song, Prof. J. Wu
School of Life Sciences and Health Engineering
Jiangnan University
Wuxi 214122, China

Contents

Supporting Tables 1-4

Supporting Figures 1-8

1. Supporting Table 1 to Table 4

Table S1. Plasmids used in this study

Plasmid	Feature	Source
pCDR	pCDR with Trc promoter, lacO operator and T7 terminator, Str[r]	Lab storage
pEM	pEM with T5 promoter, lacO operator and T7 terminator, Amp[r]	Lab storage
pCDR-*kfoF-kfoA-kfoC*	pCDR plasmid expressing the *kfoF*, *kfoA*, *kfoC* genes from *E. coli* K4	This study
pEM-*glmS*	pEM plasmid expressing the *glmS* gene from *E. coli* BL21 STAR (DE3)	This study
pEM-*glmM*	pEM plasmid expressing the *glmM* gene from *E. coli* BL21 STAR (DE3)	This study

pEM-*glmU*	pEM plasmid expressing the *glmU* gene from *E. coli* BL21 STAR (DE3)	This study
pEM-*kfoA*	pEM plasmid expressing the *kfoA* gene from *E. coli* K4	This study
pEM-*pgi*	pEM plasmid expressing the *pgi* gene from *E. coli* BL21 STAR (DE3)	This study
pEM-*pgm*	pEM plasmid expressing the *pgm* gene from *E. coli* BL21 STAR (DE3)	This study
pEM-*galU*	pEM plasmid expressing the *galU* gene from *E. coli* BL21 STAR (DE3)	This study
pEM-*kfoF*	pEM plasmid expressing the *kfoF* gene from *E. coli* K4	This study
pEM-*gltC*	pEM plasmid expressing the *gltC* gene from *E. coli* BL21 STAR (DE3)	This study
pEM-*gdhA*	pEM plasmid expressing the *gdhA* gene from *E. coli* BL21 STAR (DE3)	This study
pEM-*glnA*	pEM plasmid expressing the *glnA* gene from *E. coli* BL21 STAR (DE3)	This study
NCS	pPMB1-Prrnc-egfp-DAS, Amp^r	[7]
NA8	pPMB1-PrrnA-egfp-DAS+8, Amp^r	[7]
NA4	pPMB1-PrpsA-egfp-DAS+4, Amp^r	[7]
PL4	p15A-PrpsL-tetR-DAS+4-Ptet-mKate, Cm^r	[7]
PLD	p15A-PrpsL-tetR-GSD-Ptet-mKate, Cm^r	[7]
PLA	p15A-PrpsL-tetR-LAA-Ptet-mKate, Cm^r	[7]

Table S2. Strains used in this study

Strain	Feature	Source
E. coli JM109	General cloning host	Lab storage
E. coli K4	Wild type Serotype O5:K4 (L):H4	Lab storage
E. coli BL21 STAR (DE3) (*E. coli* F00)	ompT hsdT hsdS (rBmB) gal (DE3)	Novagen
E. coli F01	F00, Str^r, pCDR-*kfoF-kfoA-kfoC*	This study
E. coli F02	F01, Str^r, Amp^r, pEM-*gdhA*	This study
E. coli F03	F01, Str^r, Amp^r, pEM-*glnA*	This study
E. coli F04	F01, Str^r, Amp^r, pEM-*gltC*	This study
E. coli F05	F01, Str^r, Amp^r, pEM-*gdhA-glnA*	This study
E. coli F06	F05, Str^r, Amp^r, pEM-rbsl-*glmS*	This study
E. coli F07	F05, Str^r, Amp^r, pEM-rbsm-*glmS*	This study
E. coli F08	F05, Str^r, Amp^r, pEM-rbsh-*glmS*	This study
E. coli F09	F05, Str^r, Amp^r, pEM-rbsh-*glmS**	This study
E. coli F10	*E. coli* F09, Str^r, Amp^r, pEM-*glmM*	This study
E. coli F11	*E. coli* F09, Str^r, Amp^r, pEM-*glmU*	This study
E. coli F12	*E. coli* F09, Str^r, Amp^r, pEM-*kfoA*	This study
E. coli F13	*E. coli* F10, Str^r, Amp^r, pEM-*pgi*	This study
E. coli F14	*E. coli* F10, Str^r, Amp^r, pEM-*pgm*	This study
E. coli F15	*E. coli* F10, Str^r, Amp^r, pEM-*galU*	This study
E. coli F16	*E. coli* F10, Str^r, Amp^r, pEM-*kfoF*	This study
E. coli F17	*E. coli* F16, Str^r, Amp^r, pEM-*pgi*	This study
E. coli F18	*E. coli* F16, Str^r, Amp^r, pEM-*pgm*	This study
E. coli F19	*E. coli* F16, Δ*otsA*, Str^r, Amp^r	This study
E. coli F20	*E. coli* F19, Δ*glgC*, Str^r, Amp^r	This study
E. coli F21	*E. coli* F20, Δ*zwf*, Str^r, Amp^r, NA8-*zwf*	This study
E. coli F22	*E. coli* F20, Δ*murA*, Str^r, Amp^r, NA8-*murA*	This study
E. coli F23	*E. coli* F20, Δ*SucA*, Str^r, Amp^r, NA8-*SucA*	This study
E. coli F24	*E. coli* F21, Δ*murA*, Str^r, Amp^r, NA8-*murA*	This study
E. coli F25	*E. coli* F21, Δ*SucA*, Str^r, Amp^r, NA8-*SucA*	This study
E. coli F26	*E. coli* F22, Δ*SucA*, Str^r, Amp^r, NA8-*SucA*	This study
E. coli F27	*E. coli* F24, Δ*SucA*, Str^r, Amp^r, NA8-*SucA*	This study
E. coli F28	*E. coli* F27, Str^r, Amp^r, Cm^r, NA4-*glmS-M*, PLA-*kfoF*	This study
E. coli F29	*E. coli* F27, Str^r, Amp^r, Cm^r, NA4-*glmS-M*, PL4-*kfoF*	This study
E. coli F30	*E. coli* F27, Str^r, Amp^r, Cm^r, NA4-*glmS-M*, PLD-*kfoF*	This study
E. coli F31	*E. coli* F27, Str^r, Amp^r, Cm^r, NA8-*glmS-M*, PLA-*kfoF*	This study
E. coli F32	*E. coli* F27, Str^r, Amp^r, Cm^r, NA8-*glmS-M*, PL4-*kfoF*	This study
E. coli F33	*E. coli* F27, Str^r, Amp^r, Cm^r, NA8-*glmS-M*, PLD-*kfoF*	This study
E. coli F34	*E. coli* F27, Str^r, Amp^r, Cm^r, NCS-*glmS-M*, PLA-*kfoF*	This study
E. coli F35	*E. coli* F27, Str^r, Amp^r, Cm^r, NCS-*glmS-M*, PL4-*kfoF*	This study
E. coli F36	*E. coli* F27, Str^r, Amp^r, Cm^r, NCS-*glmS-M*, PLD-*kfoF*	This study
E. coli F37	*E. coli* F35, Str^r, Amp^r, Cm^r, NCS-RBSl-*glmS-M*, PL4- RBSl-*kfoF*, pCDR- RBSl-*kfoC*	This study
E. coli F38	*E. coli* F35, Str^r, Amp^r, Cm^r, NCS-RBSl-*glmS-M*, PL4- RBSl-*kfoF*, pCDR- RBSm-*kfoC*	This study

E. coli F39	*E. coli* F35, Str^r, Amp^r, Cm^r, NCS-RBSl-*glmS-M*, PL4- RBSl-*kfoF*, pCDR- RBSh-*kfoC*	This study
E. coli F40	*E. coli* F35, Str^r, Amp^r, Cm^r, NCS-RBSl-*glmS-M*, PL4-RBSm-*kfoF*, pCDR-RBSl-*kfoC*	This study
E. coli F41	*E. coli* F35, Str^r, Amp^r, Cm^r, NCS-RBSl-*glmS-M*, PL4-RBSm-*kfoF*, pCDR-RBSm-*kfoC*	This study
E. coli F42	*E. coli* F35, Str^r, Amp^r, Cm^r, NCS-RBSl-*glmS-M*, PL4-RBSm-*kfoF*, pCDR-RBSh-*kfoC*	This study
E. coli F43	*E. coli* F35, Str^r, Amp^r, Cm^r, NCS-RBSl-*glmS-M*, PL4-RBSh-*kfoF*, pCDR-RBSl-*kfoC*	This study
E. coli F44	*E. coli* F35, Str^r, Amp^r, Cm^r, NCS-RBSl-*glmS-M*, PL4-RBSh-*kfoF*, pCDR-RBSm-*kfoC*	This study
E. coli F45	*E. coli* F35, Str^r, Amp^r, Cm^r, NCS-RBSl-*glmS-M*, PL4-RBSh-*kfoF*, pCDR-RBSh-*kfoC*	This study
E. coli F46	*E. coli* F35, Str^r, Amp^r, Cm^r, NCS-RBSm-*glmS-M*, PL4-RBSl-*kfoF*, pCDR-RBSl-*kfoC*	This study
E. coli F47	*E. coli* F35, Str^r, Amp^r, Cm^r, NCS-RBSm-*glmS-M*, PL4-RBSl-*kfoF*, pCDR-RBSm-*kfoC*	This study
E. coli F48	*E. coli* F35, Str^r, Amp^r, Cm^r, NCS-RBSm-*glmS-M*, PL4-RBSl-*kfoF*, pCDR-RBSh-*kfoC*	This study
E. coli F49	*E. coli* F35, Str^r, Amp^r, Cm^r, NCS-RBSm-*glmS-M*, PL4-RBSm-*kfoF*, pCDR-RBSl-*kfoC*	This study
E. coli F50	*E. coli* F35, Str^r, Amp^r, Cm^r, NCS-RBSm-*glmS-M*, PL4-RBSm-*kfoF*, pCDR-RBSm-*kfoC*	This study
E. coli F51	*E. coli* F35, Str^r, Amp^r, Cm^r, NCS-RBSm-*glmS-M*, PL4-RBSm-*kfoF*, pCDR-RBSh-*kfoC*	This study
E. coli F52	*E. coli* F35, Str^r, Amp^r, Cm^r, NCS-RBSm-*glmS-M*, PL4-RBSh-*kfoF*, pCDR-RBSl-*kfoC*	This study
E. coli F53	*E. coli* F35, Str^r, Amp^r, Cm^r, NCS-RBSm-*glmS-M*, PL4-RBSh-*kfoF*, pCDR-RBSm-*kfoC*	This study
E. coli F54	*E. coli* F35, Str^r, Amp^r, Cm^r, NCS-RBSm-*glmS-M*, PL4-RBSh-*kfoF*, pCDR-RBSh-*kfoC*	This study
E. coli F55	*E. coli* F35, Str^r, Amp^r, Cm^r, NCS-RBSh-*glmS-M*, PL4-RBSl-*kfoF*, pCDR-RBSl-*kfoC*	This study
E. coli F56	*E. coli* F35, Str^r, Amp^r, Cm^r, NCS-RBSh-*glmS-M*, PL4-RBSl-*kfoF*, pCDR-RBSm-*kfoC*	This study
E. coli F57	*E. coli* F35, Str^r, Amp^r, Cm^r, NCS-RBSh-*glmS-M*, PL4-RBSl-*kfoF*, pCDR-RBSh-*kfoC*	This study
E. coli F58	*E. coli* F35, Str^r, Amp^r, Cm^r, NCS-RBSh-*glmS-M*, PL4-RBSm-*kfoF*, pCDR-RBSl-*kfoC*	This study
E. coli F59	*E. coli* F35, Str^r, Amp^r, Cm^r, NCS-RBSh-*glmS-M*, PL4-RBSm-*kfoF*, pCDR-RBSm-*kfoC*	This study
E. coli F60	*E. coli* F35, Str^r, Amp^r, Cm^r, NCS-RBSh-*glmS-M*, PL4-RBSm-*kfoF*, pCDR-RBSh-*kfoC*	This study
E. coli F61	*E. coli* F35, Str^r, Amp^r, Cm^r, NCS-RBSh-*glmS-M*, PL4-RBSh-*kfoF*, pCDR-RBSl-*kfoC*	This study
E. coli F62	*E. coli* F35, Str^r, Amp^r, Cm^r, NCS-RBSh-*glmS-M*, PL4-RBSh-*kfoF*, pCDR-RBSm-*kfoC*	This study
E. coli F63	*E. coli* F35, Str^r, Amp^r, Cm^r, NCS-RBSh-*glmS-M*, PL4-RBSh-*kfoF*, pCDR-RBSh-*kfoC*	This study

Table S3. Strains used in this study

Primer	Sequence (5' to 3')
KfoF-F	GAAGGAGATATACATATGGCAGATCTGATGAAAATTGCAGTTGCTGG
KfoF-R	GCAGCGGTTTCTTTACCAGACTCGAGTCAAGAGTCGACACCAAATA
KfoA-F	GCCAGCACATGGACTCGTCTACTAGTGAAATCATAAAAAATTTATTTGCTT
KfoA-R	TTTAGGTTAATTAAGCTGCGACTAGTTTAAATATAACCATTTGGGTTTTTC
KfoC-F	TATTTGGTGTCGACTCTTGACTCGAGGGAGCTTATCGACTGCACGG
KfoC-R	GCAGCGGTTTCTTTACCAGACTCGAGTTATAAATCATTCTCTATTTTTTCCCAGGT
glmS-F	ACGGATCCGCATGCGAGCTCGGTACCATGTGTGGAATTGTTGGCGC
glmS-R	GCAGCGGTTTCTTTACCAGACTCGAGTTACTCAACCGTAACCGATT
glmM-F	ACGGATCCGCATGCGAGCTCGGTACCATGAGTAATCGTAAATATTT
glmM-R	GCAGCGGTTTCTTTACCAGACTCGAGTTAAACGGCTTTTACTGCAT
glmU-F	ACGGATCCGCATGCGAGCTCGGTACCATGTTGAATAATGCTATGAG
glmU-R	GCAGCGGTTTCTTTACCAGACTCGAGTCACTTTTTCTTTACCGGAC
KfoA-F	ACGGATCCGCATGCGAGCTCGGTACCATGAATATATTAGTTACAGG

KfoA-R	GCAGCGGTTTCTTTACCAGACTCGAGTTAAATATAACCATTTGGGT
Pgi-F	ACGGATCCGCATGCGAGCTCGGTACCATGAAAAACATCAATCCAACGC
Pgi-R	GCAGCGGTTTCTTTACCAGACTCGAGTTAACCGCGCCACGCTTTAT
Pgm-F	ACGGATCCGCATGCGAGCTCGGTACCATGGCAATCCACAATCGTGC
Pgm-R	GCAGCGGTTTCTTTACCAGACTCGAGTTACGCGTTTTTCAGAACTT
KfoF-F	ACGGATCCGCATGCGAGCTCGGTACCATGAAAATTGCAGTTGCTGG
KfoF-R	GCAGCGGTTTCTTTACCAGACTCGAGTCAAGAGTCGACACCAAATA
GalU-F	ACGGATCCGCATGCGAGCTCGGTACCATGGCTGCCATTAATACG
GalU-R	GCAGCGGTTTCTTTACCAGACTCGAGTTACTTCTTAATGCCCATCT
zwf-F	GTAAAGAGGAGAAAAAGCTTGGGCCCATGGCGGTAACGCAAACAGC
zwf-R	CTGAGTAGTTCTCATCATTTGCGGCCTCAAACTCATTCCAGGAACGACCA
SucA-F	TAAAGAGGAGAAAAAGCTTGGGCCCATGCAGAACAGCGCTTTGAAAG
SucA-R	TAGTTCTCATCATTTGCGGCTTCGACGTTCAGCGCGTCAT
murA-F	AAAGAGGAGAAAAAGCTTGGGCCCATGGATAAATTTCGTGTTCAGGGGC
murA-R	TAGTTCTCATCATTTGCGGCTTCGCCTTTCACACGCTCAA
GlmSN1-F	GTAAAGAGGAGAAAAAGCTTGGGCCCATGTGTGGAATTGTTGGCGC
GlmSN1-R	CATAGTTTTCATCATTTGCCGCCTCAACCGTAACCGATTTTGCC
GlmSN2-R	TAATTTTCGTCGTTGGCAGCCTCAACCGTAACCGATTTTGCC
GlmSN3-R	TGAGTAGTTCTCATCATTTGCGGCCTCAACCGTAACCGATTTTGCC
GlmMN1-F	GCGCCGAAAGAGGAGAAAGGGCCCATGAGTAATCGTAAATATTTCGGTA
GlmMN1-R	TAGTTTTCATCATTTGCCGCAACGGCTTTTACTGCATCGG
GlmMN2-R	GTAATTTTCGTCGTTGGCAGCAACGGCTTTTACTGCATCGG
GlmMN3-R	GAGTAGTTCTCATCATTTGCGGCAACGGCTTTTACTGCATCGG
kfoFN-F	CGATTAAAGAGGAGAAAACGCGTATGAAAATTGCAGTTGCTGGTGTAG
kfoFN-R	TTATTTGATGCCTGGAGATCCTTACTCGAGTCAAGAGTCGACACCAAATAAATCTCGC
kfoFRl-F	TTAAGGAGGAATTCTTACGCGTATGAAAATTGCAGTTGCTG
kfoFRl-R	TACGCGTAAGAATTCCTCCTTAAGAATTCTTTTCTCTATCACTGATAGGGAGT
kfoFRm-F	GTCATACACAAGACGAACGAATCAGGGGGGTCAAGACGCGTATGAAAATTGCAGT
kfoFRm-R	CTTGACCCCCCTGATTCGTTCGTCTTGTGTATGACGAATTCTTTTCTCTATCACTGATAG
glmSRl-F	TAGTTTAAGGAGGAATTCTTAAGCTTGGGCCCATGTGTGG
glmSRl-R	AGCTTAAGAATTCCTCCTTAAACTAGTTTTGATCTCGGCCA
glmMRl-F	CGCCGTTAAGGAGGAATTCTTGGGCCCATGAGTAATCGTAA
glmMRl-R	GCCCAAGAATTCCTCCTTAACGGCGCTATTTTAGCTGGCA
glmSRm-F	GTCATACACAAGACGAACGAATCAGGGGGGTCAAGAAGCTTGGGCCCATGTGTGG
glmSRm-R	CTTGACCCCCCTGATTCGTTCGTCTTGTGTATGACACTAGTTTTGATCTCGGCCAA
glmMRm-F	GTCATACACAAGACGAACGAATCAGGGGGGTCAAGGGGCCCATGAGTAATCGTAA
glmMRm-R	CTTGACCCCCCTGATTCGTTCGTCTTGTGTATGACCGGCGCTATTTTAGCTGGC
glmSRh-F	CTAGTGATTAAAGAGGAGAAAAAGCTTGGGCCCATGTGTGG
glmSRh-R	GCTTTTTCTCCTCTTTAATCACTAGTTTTGATCTCGGCCAAA
glmMRh-F	GCCGGATTAAAGAGGAGAAAGGGCCCATGAGTAATCGTAA
glmMRh-R	GCCCTTTCTCCTCTTTAATCCGGCGCTATTTTAGCTGGC
kfoCRl-F	AACAATTAAGGAGGAATTCTTGAATTCATGAGTATTCTTAATCAAGCAATA
kfoCRl-R	ATTCAAGAATTCCTCCTTAATTGTTATCCGCTCACAATTCC
kfoCRm-F	GTCATACACAAGACGAACGAATCAGGGGGGTCAAGGAATTCATGAGTATTCTTAATCAAG
kfoCRm-R	CTTGACCCCCCTGATTCGTTCGTCTTGTGTATGACTTGTTATCCGCTCACAATTCCA

Table S4. A comparative analysis of chondroitin production.

Host	Strategy	Titer (g/L)	Reference
E. coli BL21 star (DE3)	Co-expressing *kfoF*, *kfoA* and *kfoC*	2.4	[2]
C. glutamicum	Co-expressing *ugd* and *glmS*	7.4	[3]
B. subtilis	Co-overexpressing *tuaD*, *glmM* and *kfoA*	7.15	[4]

B. subtilis	Overexpressing *tuaD*	5.22	[5]
C. glutamicum	Co-overexpressing *ugdA*, *kfoC* and *kfoA*, knocking out *ldh*	1.91	[6]
E. coli BL21 star (DE3)	Dynamically regulated *glmS-glmM*, *kfoF* and *kfoC*	9.2	This study

2. Supporting Figure 1 to Figure 8

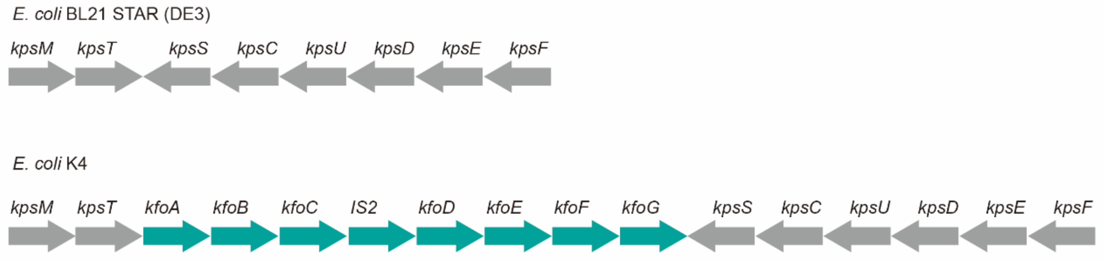

Figure S1. Polysaccharides biosynthesis gene clusters associated with chondroitin in *E. coli* BL21 STAR (DE3) and *E. coli* K4. The *kfoA*, *kfoC*, and *kfoF* genes are situated within the gene cluster of chondroitin synthesis in *E. coli* K4.

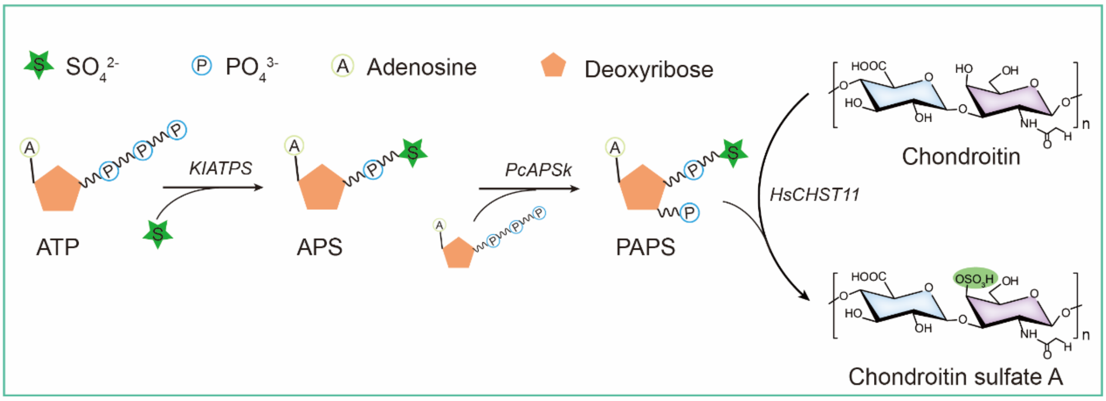

Figure S2. The cascade pathway of Chondroitin Sulfate A (CSA) synthesis in vitro. In this pathway, 3'-phosphoadenosine-5'-phosphosulfate (PAPS) serves as the sulfonyl donor, and chondroitin acts as the sulfonyl acceptor, facilitating the biosynthesis of CSA. Following our previous study, PAPS is synthesized using one molecule of SO_4^{2-} and two molecules of ATP as substrates. This process is catalyzed by ATP sulfurylase from *Kluyveromyces lactis* (*KlATPS*) and adenosine 5′-phosphosulfate kinase from *Penicillium chrysogenum* (*PcAPSK*). Then, chondroitin is sulfated using PAPS by sulfotransferases derived from *Homo sapiens* (*HsCHST*11)[6].

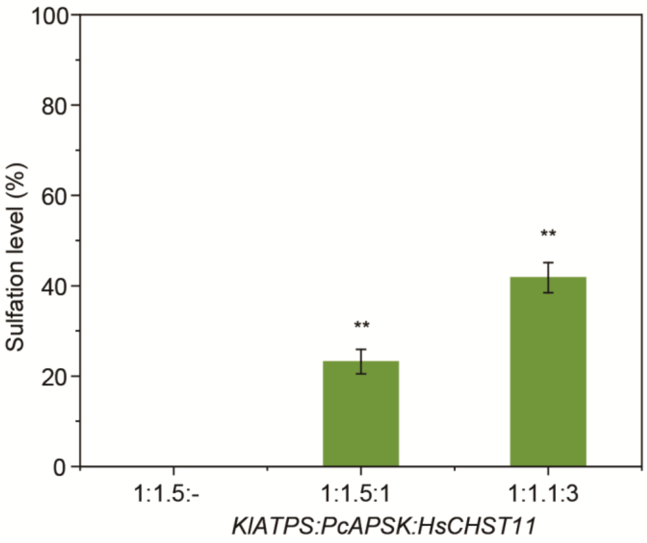

Figure S3. Sulfation levels achieved through varying catalytic ratios of KlATPS, PcAPSK, and HsCHST11.

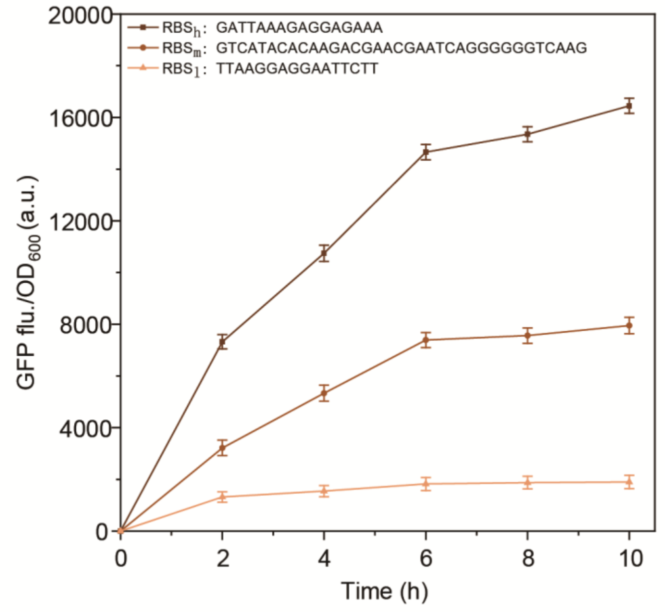

Figure S4. The fluorescence intensity of different ribosome binding sites (RBS) in *E. coli* BL21 STAR (DE3).

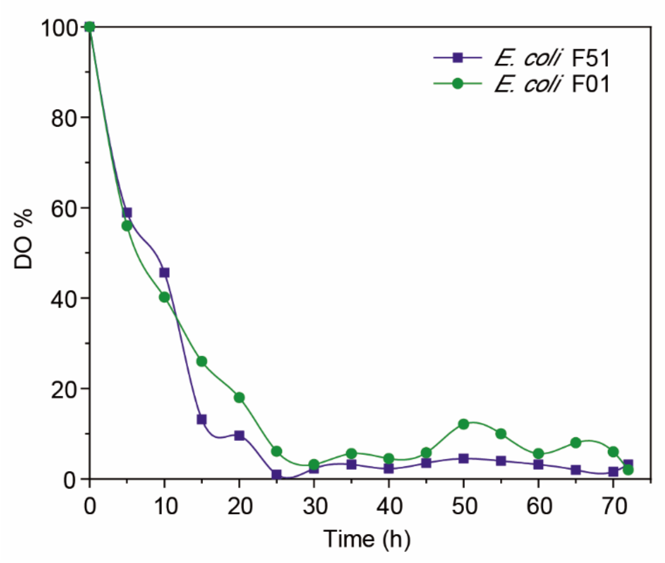

Figure S5. Dissolved Oxygen (DO) monitoring of strains *E. coli* F01 and F51 during 72 h fermentation.

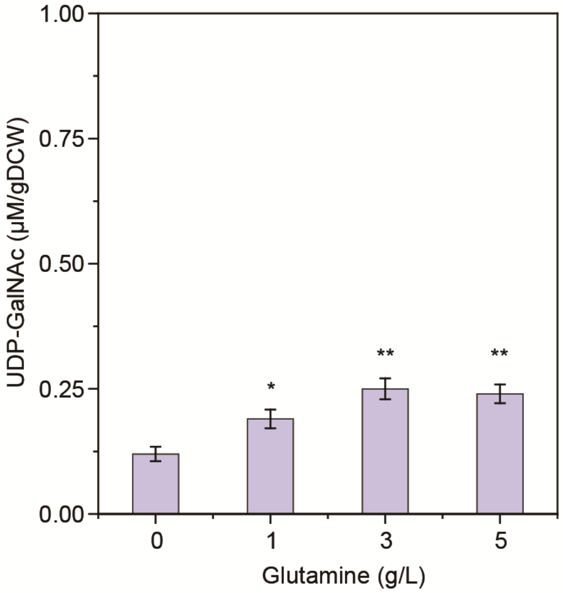

Figure S6. The effect of glutamine addition on UDP-GalNAc accumulation in *E. coli* F01.

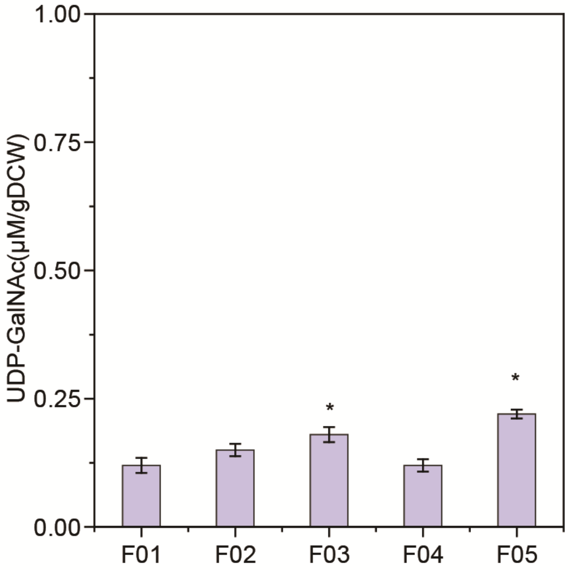

Figure S7. The effect of gene overexpression in glutamine pathway on UDP-GalNAc accumulation in different recombinant *E. coli*.

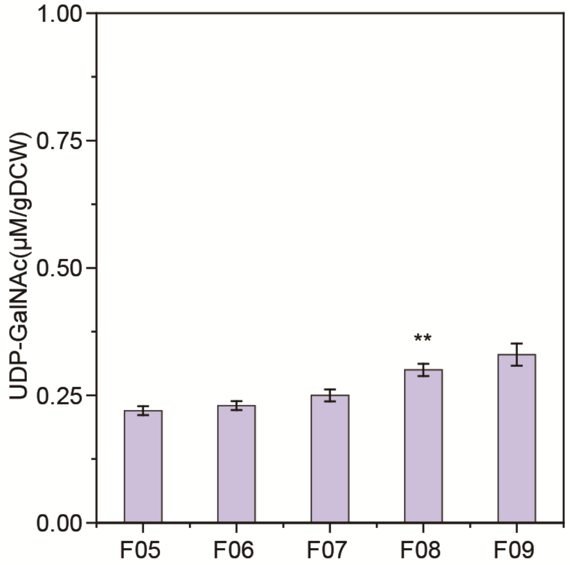

Figure S8. The effect of *glmS* overexpression on UDP-GalNAc accumulation.

3. References

[1] Q. Zhang, R. Yao, X. L. Chen, L. M. Liu, S. Q. Xu, J. H. Chen, J. Wu, *Metab Eng*. **2018**, 47, 314-322.
[2] C. Liu, X. Lv, J. Li, L. Liu, G. Du, Y. Liu, *J Agric Food Chem*. **2022**, 70, 15859-15868.
[3] M. Zhao, D. Huang, X. Zhang, M. A. G. Koffas, J. Zhou, Y. Deng, *Metab Eng* **2018**, 47, 254-262.
[4] M. Oleksy, E. Klewicka, *Probiotics Antimicrob Proteins*. **2017**, 9, 425-434.
[5] Q. Liu, J. Zhang, X. X. Wei, S. P. Ouyang, Q. Wu, G. Q. Chen, *Appl Microbiol Biotechnol* **2008**, 77, 1297-1304.
[6] H. Liu, W. Wei, Z. Pang, S. Gu, W. Song, C. Gao, X. Chen, J. Liu, L. Guo, J. Wu, L. Liu, *Biotechnol Bioeng*. **2023**, 120, 1784-1796.
[7] J. Hou, C. Gao, L. Guo, J. Nielsen, Q. Ding, W. Tang, G. Hu, X. Chen, L. Liu, *Metab Eng* **2020**, 61, 47-57.
